# Association of short-term air pollution with risk of major adverse cardiovascular event mortality and modification effects of lifestyle in Chinese adults

**DOI:** 10.1265/ehpm.24-00340

**Published:** 2025-05-13

**Authors:** Wendi Xiao, Xin Yao, Yinqi Ding, Junpei Tao, Canqing Yu, Dianjianyi Sun, Pei Pei, Ling Yang, Yiping Chen, Huaidong Du, Dan Schmidt, Yaoming Zhai, Junshi Chen, Zhengming Chen, Jun Lv, Liqiang Zhang, Tao Huang, Liming Li

**Affiliations:** 1 Department of Epidemiology and Biostatistics, School of Public Health, Peking University, Beijing 100191, China; 2 State Key Laboratory of Remote Sensing Science, Faculty of Geographical Science, Beijing Normal University, Beijing 100085, China; 3 Peking University Center for Public Health and Epidemic Preparedness & Response, Beijing 100191, China; 4 Key Laboratory of Epidemiology of Major Diseases (Peking University), Ministry of Education, Beijing 100191, China; 5 Clinical Trial Service Unit & Epidemiological Studies Unit (CTSU), Nuffield Department of Population Health, University of Oxford, Oxford, United Kingdom; 6 Qingdao Center for Disease Control and Prevention, Qingdao 266033, China; 7 China National Center for Food Safety Risk Assessment, Beijing 100022, China; 8 State Key Laboratory of Vascular Homeostasis and Remodeling, Peking University, Beijing 100191, China

**Keywords:** Ambient air pollution, Short-term, Case-crossover, MACE, Mortality, China, CKB

## Abstract

**Background:**

Previous evidence showed that ambient air pollution and cardiovascular mortality are related. However, there is a lack of evidence towards the modification effect of long-term lifestyle on the association between short-term ambient air pollution and death from cardiovascular events.

**Method:**

A total of 14,609 death from major adverse cardiovascular events (MACE) were identified among the China Kadoorie Biobank participants from 2013 to 2018. Ambient air pollution exposure including particulate matter 2.5 (PM_2.5_), SO_2_, NO_2_, CO, and O_3_ from the same period were obtained from space-time model reconstructions based on remote sensing data. Case-crossover design and conditional logistic regression was applied to estimate the effect of short-term exposure to air pollutants on MACE mortality.

**Results:**

We found MACE mortality was significantly associated with PM_2.5_ (relative percent increase 2.91% per 10 µg/m^3^ increase, 95% CI 1.32–4.53), NO_2_ (5.37% per 10 µg/m^3^ increase, 95% CI 1.56–9.33), SO_2_ (6.82% per 10 µg/m^3^ increase, 95% CI 2.99–10.80), and CO (2.24% per 0.1 mg/m^3^ increase, 95% CI 1.02–3.48). Stratified analyses indicated that drinking was associated with elevated risk of MACE mortality with NO_2_ and SO_2_ exposure; physical inactivity was associated with higher risk of death from MACE when exposed to PM_2.5_; and people who had balanced diet had lower risk of MACE mortality when exposed to CO and NO_2_.

**Conclusions:**

The study results showed that short-term exposure to ambient PM_2.5_, NO_2_, SO_2_, and CO would aggravate the risk of cardiovascular mortality, yet healthy lifestyle conduct might mitigate such negative impact to some extent.

**Supplementary information:**

The online version contains supplementary material available at https://doi.org/10.1265/ehpm.24-00340.

## Introduction

Cardiovascular diseases (CVDs) are the leading cause of morbidity and mortality worldwide [1]. According to the Global Burden Disease, Injuries, and Risk Factors Study (GBD), the total disability-adjusted life-years (DALYs) due to ischemic heart disease (IHD) and stroke in 2021 were estimated to be 188.3 million and 160.4 million, respectively [2], posing a major public health concern. Among factors that contributing to CVD, the air pollution was the top environmental risk trigger [3], especially in middle- to low-income countries [4]. The air pollution problem in China concerned mainly particulate matter (PM), sulfur oxide (SO_X_), nitrogen oxide (NO_X_), carbon oxide (CO_X_), and ozone (O_3_) [5, 6]. Furthermore, the air pollution is graver in China because the level of pollutants was well above the thresholds as instructed in guidelines [7]. These pollutant particles may inflict oxidative stress on the respiratory tract cells after inhalation and can be transmitted to the systemic circulation, causing inflammation and subsequent CV events [8, 9].

Major adverse cardiovascular events (MACE) including myocardial infarction and stroke is among the top disease burdens in China, and there is an emerging body of evidence elucidating the association between short-term exposure to ambient air pollution and MACE, both fatal and non-fatal [10–12]. Vulnerable sub-population were found including elderly and people with previous diagnosed cardiac problems [4]. However, there is a lack of study focusing on the modifying effect from lifestyle factors, given that individual-level lifestyles were unavailable for most previous studies investigating short-term air pollutant exposure. The modifying effect of lifestyle on long-term effects of PM_2.5_ had been reported in cohort study design, which had been verified in large Chinese cohorts [13, 14]. Thus, it is to our interest whether lifestyle might have similar effect modification in the association between short-term air exposure and CVD outcomes.

In this study, we utilized a national-representative sample of 14,609 death with MACE as the underlying cause in China from 2013 to 2018 and investigated the association between ambient air pollution (PM_2.5_, SO_2_, CO, NO_2_, O_3_) and odds of death from MACE using the case-crossover design. We further studied individual-level lifestyles (i.e., BMI, dietary pattern, physical activity level, drinking, and smoking habit) and examined their potential modifying effect in stratified analyses.

## Methods

### Study population

The China Kadoorie Biobank (CKB) was a population-based, cohort study initiated in 2004 with a total of 512,715 Chinese adults aged 30 to 79 enrolled. Detailed information on CKB design and data were reported in [15] and [16]. In brief, participants with aids from trained interviewer filled out electronic questionnaire that covered aspects including socio-demographic characteristics, lifestyle, and history of medication [17]. Informed consents were obtained from all participants. The study was approved by both the Ethical Review Committee of the Chinese Center for Disease Control and Prevention (Beijing, China: 005/2004) and the Oxford Tropical Research Ethics Committee, University of Oxford (UK: 025-04).

### Air pollution data

Air pollution data were accessed through the China High Air Pollutants (CHAP), which provided high-quality data of short-term exposure to seven major air pollutants, chemical composition of PM_2.5_, and ambient polycyclic aromatic hydrocarbons [18–20]. To measure air pollutants from 2013 to 2018, we extracted daily grid data of PM_2.5_, NO_2_, SO_2_, CO (measured as daily average), and O_3_ (measured as maximum 8 h average) at 1 km × 1 km spatial resolution from CHAP and used a bilinear interpolation resampling algorithm to expand the grid at 5 km × 5 km resolution. We then calculated the region-level average concentration by summing up each grid concentration within the region boundary and get the daily average for each CKB study site region respectively.

Meteorological factors including temperature, relative humidity, and wind speed were collected from the fifth generation of the European ReAnalysis (ERA5)-Land reanalysis data set from 2013 to 2018 [21].

### Outcome

The outcome of interest in study was mortality with MACE as the underlying cause of death. MACE was defined by the International Statistical Classification of Disease and Related Health Problems, 10^th^ Revision (ICD-10) code including I21–I25 (ischaemic heart disease), I60–I61, I63–I64 (any stroke), and other vascular disease codes (I00–I20, I27–I59, I62, I65–I88, I95–I99) [22]. The CKB were linked with the National Mortality Surveillance System and the diagnosis of the underlying cause of death for each case were of high reliability [23]. From CKB follow-up, we identified a total of 14,609 participants who died from MACE between 2013–2018.

### Study design

The study employed a time-stratified case-crossover design to investigate the association of ambient air pollutants (PM_2.5_, NO_2_, SO_2_, CO, and O_3_) with MACE mortality. Case-crossover study is widely used to study the transient effects of environmental factors on health outcomes [24, 25]. The core idea behind the case-crossover design is to assess the air exposure level on the day that death from MACE happens (the case day) against the air exposure levels on surrounding days (control days). By doing so, the study seeks to uncover potential differences in the air exposure levels that might explain variations in the occurrence of MACE mortality.

### Sampling and implementation

In this study, the date of MACE mortality was referred to as the case day, and the control days were selected such that they share the same year, month, and day of the week with the case days. For example, if one CKB participant died from MACE on March 3, 2012 (Saturday), then March 3, 2012 would be the case day, March 2012 would be the time stratum, and all other Saturdays in the time stratum, namely March 10, 17, 24, and 31, would be the control days [26]. A total of 14,609 CKB participants who died from MACE were identified, corresponding to 14,609 case days. For each case day, 3 to 4 control days were selected, resulting in a cumulative total of 49,633 control days for comparative analysis. The advantage is that each participant acts as his or her own reference with variation only in the exposure of interest, thus the design controls for potential confounding factors such as individual level demographics as well as seasonal effects.

### Statistical analysis

The conditional logistic regression model was used to estimate odds ratios of MACE mortality for a 10 µg/m^3^ increase of PM_2.5_, NO_2_, SO_2_, and O_3_, as well as for a 0.1 mg/m^3^ increase of CO. We tested models with different moving averages of air pollutants and the period with least Akaike information criterion (AIC) was displayed. The models were adjusted with a 7-day moving averages of daily temperature, relative humidity, and wind speed, which were included in the model as natural cubic splines (degrees of freedom = 5). In order to estimate the concentration-response curve for each air pollutant, natural cubic splines (degrees of freedom = 5) were used. We reported the relative percent changes in death from MACE as (odds ratio − 1) × 100%, given that the odds ratios were generally very small in scale.

We repeated analyses in stratified groups based on both demographic and lifestyle factors [10, 27].

In addition, a composite healthy lifestyle score is calculated by assigning 1 point for each healthy behaviour, with the total score ranging from 0 to 5 based on the sum of individual lifestyle components (BMI, diet, smoking, drinking, or physical activeness). Stratified analysis by the healthy lifestyle score was then performed. The effects were estimated for each stratum respectively, and the difference was assessed using two-sample z tests [28, 29].

Sensitivity analyses were conducted to verify the robustness of the results. We fitted two-pollutant model by including additional pollutants and compared the nested models using likelihood ratio test. All p-values reported were two-sided and were considered statistically significant if smaller than 0.05. The data preparation, modeling, and visualization were performed using the statistical software R (version 4.3.1).

## Results

### Characteristic of sample population and air pollutants

A total of 14,609 MACE death was recorded in CKB from 2013 to 2018, out of which 53.3% were male, 15.8% died before 65, and 38.8% were urban residents (Table 1). The mean concentration of ambient air pollutant exposure on case days were 47.6 µg/m^3^ for PM_2.5_, 29.9 µg/m^3^ for SO_2_, 30.0 µg/m^3^ for NO_2_, 83.2 µg/m^3^ for O_3_, and 1.10 mg/m^3^ for CO; the mean temperature, relative humidity, and wind speed were 12.6 °C, 71.5%, and 2.1 m/s, respectively (Table 2). There was no large difference between case and control days in the distribution of air exposure. The air pollutant exposures were positively correlated for PM_2.5_, SO_2_, NO_2_, and CO, whereas O_3_ was negatively correlated with all other air pollutants (all p value <0.05, Supplementary Fig. 1). The temporal trends of case and control days by year and month are shown in Supplementary Fig. 2.

**Table 1:** Characteristic of participants with death from MACE (n = 14,609) in CKB, 2013–2018.

**Characteristics**	**Statistic**
Age at death, median (IQR)	76 (69–81)
Gender, n (%)	
Male	7786 (53.3)
Female	6823 (46.7)
Education level, n (%)	
≥middle school	4154 (28.4)
≤primary school	10455 (71.6)
Marriage status, n (%)	
Married	11406 (78.1)
Other status	3203 (21.9)
Residence, n (%)	
Urban	5666 (38.8)
Rural	8943 (61.2)
Season, n (%)	
Warm (May–October)	6540 (44.8)
Cold (November–April)	8069 (55.2)

**Table 2:** Distribution of ambient air pollutants and meteorological conditions on control and case days.

	**Mean**	**SD**	**Percentile**				

			**5th**	**25th**	**50th**	**75th**	**95th**
Case day (n = 14,609)							
PM_2.5_, µg/m^3^	47.6	27.1	17.6	28.3	40.8	58.8	105.8
CO, mg/m^3^	1.1	0.5	0.4	0.7	1.0	1.4	2.1
SO_2_, µg/m^3^	29.9	20.3	8.3	14.5	23.9	39.6	72.9
NO_2_, µg/m^3^	30.0	14.4	12.6	19.2	26.9	37.7	59.6
O_3_, µg/m^3^	83.2	32.8	38.8	58.6	77.9	103.4	143.3
Temperature, °C	12.6	11.4	−7.0	4.8	13.9	21.7	28.4
Relative humidity, %	71.5	16.2	40.1	61.9	74.7	83.8	92.5
Wind speed, m/s	2.1	1.4	0.4	1.0	1.8	2.8	4.8
Control day (n = 49,633)							
PM_2.5_, µg/m^3^	47.0	26.6	17.2	28.1	40.4	58.0	103.9
CO, mg/m^3^	1.1	0.5	0.5	0.7	1.0	1.3	2.1
SO_2_, µg/m^3^	29.7	20.2	8.3	14.5	23.8	39.0	72.9
NO_2_, µg/m^3^	29.9	14.3	12.6	19.1	26.8	37.5	58.7
O_3_, µg/m^3^	83.3	33.2	38.6	58.5	77.8	103.2	144.7
Temperature, °C	12.7	11.4	−6.7	4.9	14.0	21.7	28.3
Relative humidity, %	71.5	16.2	40.1	61.8	74.9	83.9	92.5
Wind speed, m/s	2.1	1.4	0.4	1.0	1.8	2.8	4.8

### Short-term ambient air pollution associated with MACE mortality

**Fig. 1:**
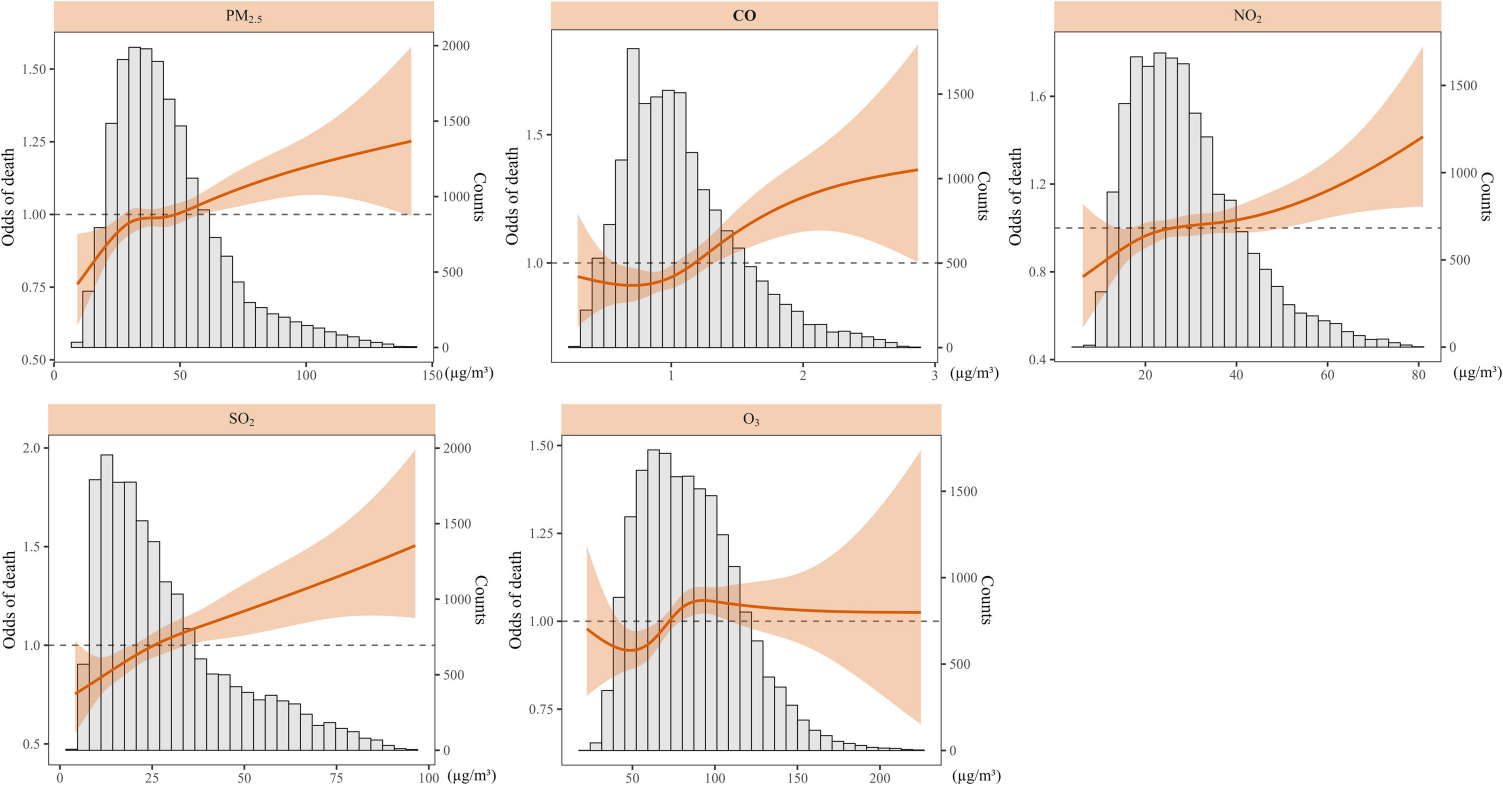
Concentration-response curve between short-term exposure to air pollution and MACE mortality.

The results showed that short-term exposure to ambient air pollution including PM_2.5_, NO_2_, SO_2_, and CO were associated with an increased risk of mortality from MACE, which were small but statistically significant (Fig. 1). The estimated relative percent increase in odds of MACE mortality were 2.91% (95% CI: 1.32–4.53) per 10 µg/m^3^ increase in PM_2.5_, 5.37% (1.56–9.33) per 10 µg/m^3^ increase in NO_2_, 6.82% (2.99–10.80) per 10 µg/m^3^ increase in SO_2_, and 2.24% (1.02–3.48) per 0.1 mg/m^3^ increase in CO. The evidence of association between O_3_ and MACE mortality risk was not significant (1.00%, 95% CI: −0.36 to 2.38 per 10 µg/m^3^ increase). The AIC of each model with air pollution exposure using different moving averages were displayed in Supplementary Fig. 3.

The concentration-response curves were also plotted displaying the association between PM_2.5_, NO_2_, SO_2_, CO and the odds of death from MACE. The results showed a concave-down pattern for PM_2.5_ and CO, with rapid increase of odds at lower concentrations and then plateaued at higher concentrations. The association between SO_2_ and NO_2_ and odds of death showed a rather linear relationship.

### Stratified analyses

**Fig. 2:**
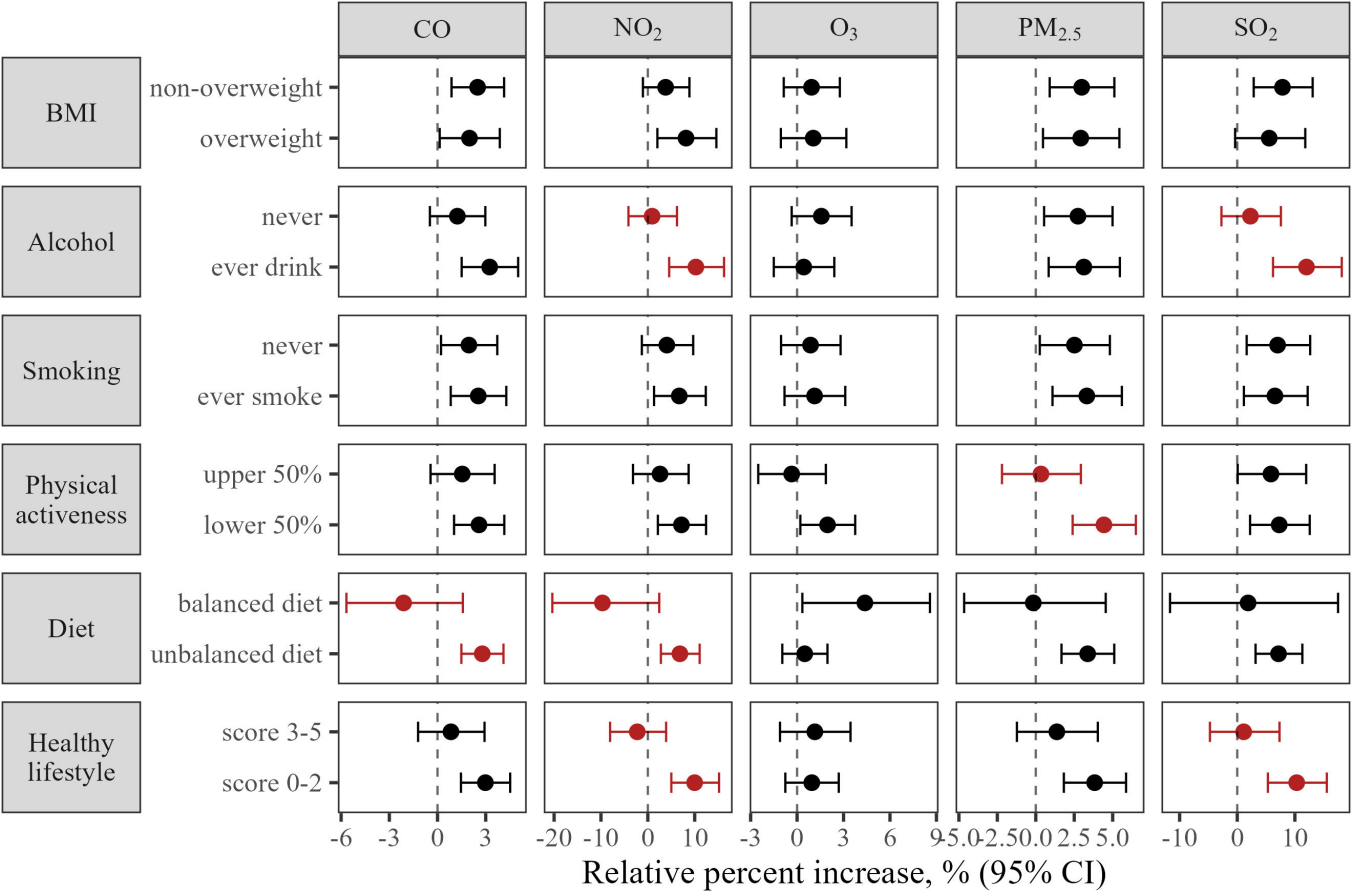
Subgroup analyses of association between short-term exposure to air pollution and MACE mortality. BMI: body mass index, overweight was defined as BMI ≥24; physical active was defined as in the upper half of metabolic equivalent of task in population with same age and sex; balanced diet was defined as daily consumption of fresh vegetable and fruit and weekly consumption of meat, fish, or poultry.

**Fig. 3:**
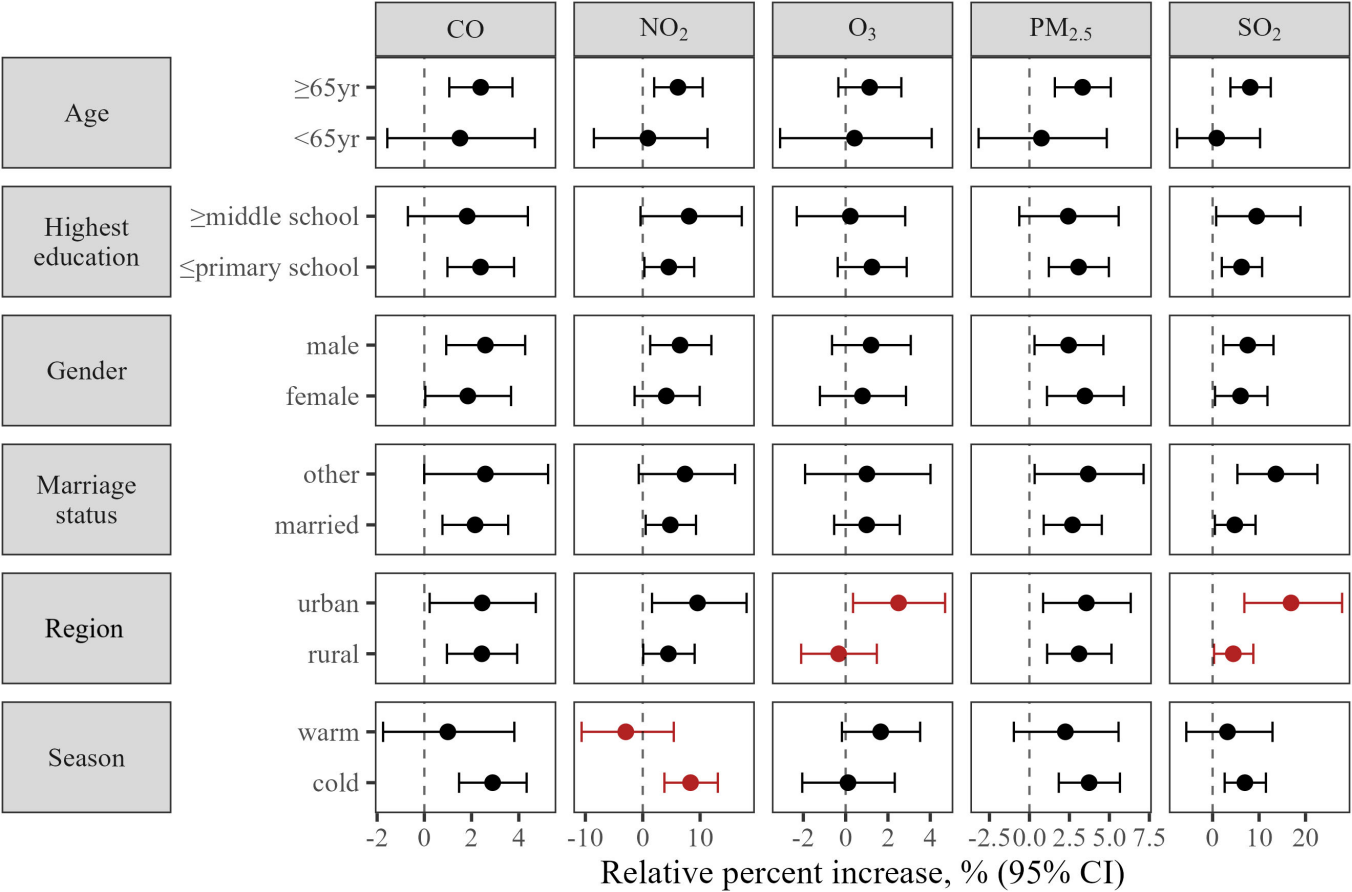
Subgroup analyses of association stratified by socio-demographic factors. Warm season included May to October, cold season included November to April.

We repeated analyses in subgroups stratified by demographic and lifestyle factors and results were summarized in Fig. 2 and 3. We observed urban participants had higher risk of MACE mortality when exposed to SO_2_ compared with rural participants. We also found NO_2_ exposure was associated with elevated risk of MACE mortality in cold season but not in warm season. There was evidence of heterogeneity, in addition, between subgroups stratified by drinking, diet, and physical activeness. It was shown that participants who never drank had lower risk of MACE death when exposed to NO_2_ or SO_2_, participants with balanced diet had lower risk of mortality when exposed to CO or NO_2_, and participants who were physically active had lower risk of death when exposed to PM_2.5_. Better lifestyle conduct as reflected by higher healthy lifestyle score was associated with lower risk of mortality for NO_2_ and SO_2_ exposure. No statistically significant difference was found between strata by age, sex, education, marital status, BMI, or smoking. Association of short-term exposure to air pollution and risk of MACE mortality by demographic and lifestyle factors were reported in detail in Supplementary Table 1 and 2.

### Sensitivity analyses

When testing the association between risk of death from MACE and air pollutants using different lag days and moving averages of exposure, we observed estimates of odds ratio with similar magnitudes and same direction, though some were not statistically significant, indicating that the results were consistent and robust in the analysis (Supplementary Fig. 4). We further adjusted for each air pollutant in the two-pollutant models and tested the robustness of results (Table 3). After model adjustment, we observed none or small heterogeneity towards the null hypothesis after the addition of new pollutant for estimated effects of PM_2.5_, CO, NO_2_, and SO_2_.

**Table 3:** Estimated percent change in odds of death from MACE using 2-pollutant models.

**Air pollutant**	**Model**	**Relative percent change (95% CI)**	**P for heterogeneity**
PM_2.5_	single	2.91 (1.32–4.53)	
	+CO	1.88 (−0.27–4.09)	0.206
	+NO_2_	2.36 (0.39–4.37)	0.795
	+SO_2_	1.99 (0.22–3.78)	0.275
	+O_3_	3.02 (1.42–4.65)	0.043
CO	single	2.24 (1.02–3.48)	
	+PM_2.5_	1.36 (−0.30–3.05)	0.404
	+NO_2_	2.07 (0.32–3.85)	0.812
	+SO_2_	1.41 (−0.07–2.91)	0.421
	+O_3_	2.40 (1.16–3.65)	0.032
NO_2_	single	5.37 (1.56–9.33)	
	+CO	1.13 (−4.10–6.65)	0.082
	+PM_2.5_	2.00 (−2.56–6.78)	0.130
	+SO_2_	1.08 (−3.79–6.21)	0.226
	+O_3_	5.34 (1.49–9.33)	0.063
SO_2_	single	6.82 (2.99–10.80)	
	+CO	4.39 (−1.50–9.14)	0.137
	+NO_2_	6.25 (1.33–11.41)	0.604
	+PM_2.5_	4.97 (0.82–9.28)	0.128
	+O_3_	6.84 (2.99–10.83)	0.059
O_3_	single	1.00 (−0.36–2.38)	
	+CO	1.23 (−0.16–2.64)	0.003
	+NO_2_	0.86 (−0.53–2.27)	0.098
	+SO_2_	0.66 (−0.74–2.08)	0.021
	+PM_2.5_	0.76 (−0.62–2.16)	0.012

## Discussion

In this study, we investigated the association between short-term exposure to air pollutants and risk of death from MACE in a nationally representative sample of Chinese population. We demonstrated evidence of association between short-term exposure to PM_2.5_, NO_2_, SO_2_, and CO with increased risk of MACE mortality and the results were largely consistent in sensitivity analyses. Stratified analyses highlighted that participant with risky lifestyle would have elevated risk of MACE mortality when shortly exposed to ambient air pollutants compared with people who adapted healthy lifestyles.

To the best of our knowledge, this was among first few studies on the effect modification from lifestyle factors on the association between short-term ambient air pollution exposure and CVD events. With detailed information collection on lifestyle, we were able to show that that people who never drank had smaller risk of death when exposed to NO_2_, and SO_2_ compared with the drinking population, and people who were physically active had smaller risk of death compared with people who lacked physical activeness when exposed to PM_2.5_, and people who had balanced diet would have lower risk when exposed to CO and NO_2_. Compared with effect modification on long-term air pollution exposure, we observed similar benefits of healthy lifestyles, thus an important primary prevention mean against air pollution-related cardiovascular events [30].

In China, the burden of cardiovascular diseases and the air pollution problem co-exist. The CVDs are the top cause of death in China with increasing mortality each year. The air quality in China has been greatly improved since the implementation of the clean air policy in 2013, thanks to the large decrease in anthropogenic emissions [31]. As we observed from air pollutant data, the median of daily PM_2.5_ decreased from 47.9 µg/m^3^ in 2013 to 30.8 µg/m^3^ in 2018, and the median of daily SO_2_ dropped from 30.8 µg/m^3^ in 2013 to 13.7 µg/m^3^ in 2018. However, the air pollution of PM_2.5_ still exceeds the national air quality guideline (35 µg/m^3^ for 24-h average) as well as the WHO Air Quality Guideline (15 µg/m^3^ for 24-h average) [7]. A recent comment on PM_2.5_ and health outcome relationship pointed out that the odds of death from CVDs increased more rapidly when exposed to PM_2.5_ at lower levels, thus there is still room for the air quality to improve and to reduce the negative impact on public health [32].

There is a growing body of evidence of association between short-term exposure to ambient air pollutants and CVD related events and our results largely corroborated with previous findings. In 2021, Liu et al. reported short-term exposure to PM_2.5_, PM_10_ and NO_2_ were significantly associated with an elevated odds of myocardial infarction mortality in a Chinese population [10]. Another report in 2013 verified the association between PM_2.5_ and odds of death from MI in another group of Chinese people, and found that extreme temperature and PM_2.5_ synergistically affected MI mortality. In terms of risk of stroke mortality, Li et al. conducted a case-crossover study including 15 northern China cities to assess the association between air pollutant exposure and stroke hospitalization and found strong association between stroke association with SO_2_, NO_2_, and PM_10_ [11]. The magnitude of effects was comparable for reported estimates of odds of death, which often constitutes of smaller effects for case-crossover design, whereas estimates from cross-sectional study investigating the long-term effects of air pollutants are usually larger in magnitude. For example, a study on long-term exposure to PM_2.5_ and its association with CVDs in China reported an estimated effect of HR at 1.251 (95% CI 1.220 to 1.283) [13], which is larger in magnitudes than the reported estimates in case-crossover studies. These made sense since short-term exposure only place the population in danger for a short duration of time. Interestingly, we found a piece of evidence of association between long-term exposure to O_3_ and CVDs in China (HR 1.31, 95% CI 1.22 to 1.42) [33]. However, in our current study we did not uncover such association with short-term exposure and further research may be needed for verification.

### Strength and limits

The study has several strengths. The nationwide cohort allowed us to study the modification effect of lifestyle factors on the association between short-term air pollution exposure and risk of death from MACE, whereas other studies often collected the data from death surveillance sites and lacked the piece of information for lifestyle on the individual level. Secondly, the utilization of case-crossover design prevented the confounding effects of possible time-invariant factors since each participant acted as his or her own control, giving less biased estimates.

There are several limitations of this study. The study measured air pollution exposure on the city level, instead of on the individual scale. Thus, there might be measurement mis-classification in air pollution exposure and the estimates would be biased towards null. Nevertheless, personal-level exposure may be hard to obtain since implementation of personal portable device was not feasible due to high cost. Considering that people may commute every day, the city-level exposure may be a suitable compromise to use. Another limitation is that there may still be additional confounding factors that are potentially related with cardiovascular death which we did not measure, such as time-varying data of organic aerosol. The total number of deaths was relatively small compared with previous research; thus, the statistical power might be limited to find significant association for subgroups whose case number were small. Finally, the study was carried out in a nationally representative Chinese sample, therefore, the result was comparable to other research on Chinese population but not to be generalized to other populations without caution.

## Conclusion

In conclusion, we found a significant relationship between ambient air pollution and risk of MACE mortality in China, in which short-term exposure to PM_2.5_, NO_2_, SO_2_, and CO was associated with elevated odds of death from MACE. We also found that keeping physically active and avoiding alcohol might alleviate such detrimental effects. These findings corroborated the current evidence and could potentially be used for policy making as well as to draw attention from the general population to promote desired lifestyles.

## Abbreviations

AIC: Akaike information criterion
MACE: major adverse cardiovascular events
PM_2.5_: particulate matter 2.5
CVD: Cardiovascular disease
GBD: Global Burden Disease, Injuries, and Risk Factors Study
DALY: total disability-adjusted life-year
IHD: ischemic heart disease
BMI: body mass index
CKB: China Kadoorie Biobank
CHAP: China High Air Pollutants
ERA5: European ReAnalysis
ICD-10: International Statistical Classification of Disease and Related Health Problems, 10th Revision (ICD-10)
CI: confidence interval
HR: hazard ratio

## Supplementary information

**Additional file 1:** Members of the China Kadoorie Biobank collaborative group.

**Additional file 2:** **Supplementary Figure 1.** Correlation between air pollutants on case and control days.

**Additional file 3:** **Supplementary Figure 2.** The temporal trends of case and control days by year and month.

**Additional file 4:** **Supplementary Figure 3.** Akaike information criterion (AIC) for models with different moving averages of air pollutants.

**Additional file 5:** **Supplementary Figure 4.** Relative percent increase in risk of MACE mortality associated with air pollutants using different lag days and moving averages.

**Additional file 6:** **Supplementary Table 1.** Subgroup analyses of association between short-term exposure to air pollution and MACE mortality. **Supplementary Table 2.** Subgroup analyses of association stratified by socio-demographic factors.

## Declarations

### Availability of data and material

Air pollution data were obtained from https://weijing-rs.github.io/product.html. The CKB data access policy and procedures are available at https://www.ckbiobank.org.
