# Supplementary material for: Association of short-term air pollution with risk of major adverse cardiovascular event mortality and modification effects of lifestyle in Chinese adults
Source: Environ Health Prev Med. 2025 May 13;30:38. doi: 10.1265/ehpm.24-00340 (PMC12086099; doi:10.1265/ehpm.24-00340)
Supplement: Supplementary file 1 — Additional file 1: Members of the China Kadoorie Biobank collaborative group. [file ehpm-30-038-s001.docx]

**Members of the China Kadoorie Biobank collaborative group:**

**International Steering Committee:** Junshi Chen, Zhengming Chen (PI), Robert

Clarke, Rory Collins, Liming Li (PI), Jun Lv, Richard Peto, Robin Walters.

**International Co-ordinating Centre, Oxford:** Daniel Avery, Maxim Barnard, Derrick

Bennett, Ruth Boxall, Ka Hung Chan, Yiping Chen, Zhengming Chen, Charlotte

Clarke, Johnathan Clarke; Robert Clarke, Huaidong Du, Ahmed Edris Mohamed,

Hannah Fry, Simon Gilbert, Pek Kei Im, Andri Iona, Maria Kakkoura, Christiana

Kartsonaki, Kshitij Kolhe, Hubert Lam, Kuang Lin, James Liu, Mohsen Mazidi, Iona

Millwood, Sam Morris, Qunhua Nie, Alfred Pozarickij, Maryam Rahmati, Paul Ryder,

Dan Schmidt, Becky Stevens, Iain Turnbull, Robin Walters, Baihan Wang, Lin Wang,

Neil Wright, Ling Yang, Xiaoming Yang, Pang Yao.

**National Co-ordinating Centre, Beijing:** Xiao Han, Can Hou, Qingmei Xia, Chao

Liu, Jun Lv, Pei Pei, Dianjianyi Sun, Canqing Yu, Lang Pan.

**10 Regional Co-ordinating Centres:**

**Qingdao CDC:** Zengchang Pang, Ruqin Gao, Shanpeng Li, Haiping Duan, Shaojie

Wang, Yongmei Liu, Ranran Du, Yajing Zang, Liang Cheng, Xiaocao Tian, Hua

Zhang, Yaoming Zhai, Feng Ning, Xiaohui Sun, Feifei Li. **Licang CDC:** Silu Lv,

Junzheng Wang, Wei Hou. **Heilongjiang Provincial CDC:** Wei Sun, Shichun Yan,

Xiaoming Cui. **Nangang CDC:** Chi Wang, Zhenyuan Wu,Yanjie Li, Quan Kang.

**Hainan Provincial CDC:** Huiming Luo, Tingting Ou. **Meilan CDC:** Xiangyang

Zheng, Zhendong Guo, Shukuan Wu, Yilei Li, Huimei Li. **Jiangsu Provincial CDC:**

Ming Wu, Yonglin Zhou, Jinyi Zhou, Ran Tao, Jie Yang, Jian Su. **Suzhou CDC:**

Fang Liu, Jun Zhang, Yihe Hu, Yan Lu, Liangcai Ma, Aiyu Tang, Shuo Zhang,

Jianrong Jin, Jingchao Liu. **Guangxi Provincial CDC:** Mei Lin, Zhenzhen Lu.

**Liuzhou CDC:** Lifang Zhou, Changping Xie, Jian Lan,Tingping Zhu,Yun Liu, Liuping

Wei, Liyuan Zhou, Ningyu Chen, Yulu Qin, Sisi Wang. **Sichuan Provincial CDC:**

Xianping Wu, Ningmei Zhang, Xiaofang Chen, Xiaoyu Chang. **Pengzhou CDC:**

Mingqiang Yuan, Xia Wu, Xiaofang Chen, Wei Jiang, Jiaqiu Liu, Qiang Sun. **Gansu**

**Provincial CDC:** Faqing Chen, Xiaolan Ren, Caixia Dong. **Maiji CDC:** Hui Zhang,

Enke Mao, Xiaoping Wang, Tao Wang, Xi zhang. **Henan Provincial CDC:** Kai Kang,

Shixian Feng, Huizi Tian, Lei Fan. **Huixian CDC:** XiaoLin Li, Huarong Sun, Pan He,

Xukui Zhang. **Zhejiang Provincial CDC:** Min Yu, Ruying Hu, Hao Wang.

**Tongxiang CDC:** Xiaoyi Zhang, Yuan Cao, Kaixu Xie, Lingli Chen, Dun Shen.

**Hunan Provincial CDC:** Xiaojun Li, Donghui Jin, Li Yin, Huilin Liu, Zhongxi Fu.

**Liuyang CDC:** Xin Xu, Hao Zhang, Jianwei Chen,Yuan Peng, Libo Zhang, Chan Qu.
