# Supplementary figures and images for: Association of short-term air pollution with risk of major adverse cardiovascular event mortality and modification effects of lifestyle in Chinese adults
Source: Environ Health Prev Med. 2025 May 13;30:38. doi: 10.1265/ehpm.24-00340 (PMC12086099; doi:10.1265/ehpm.24-00340)

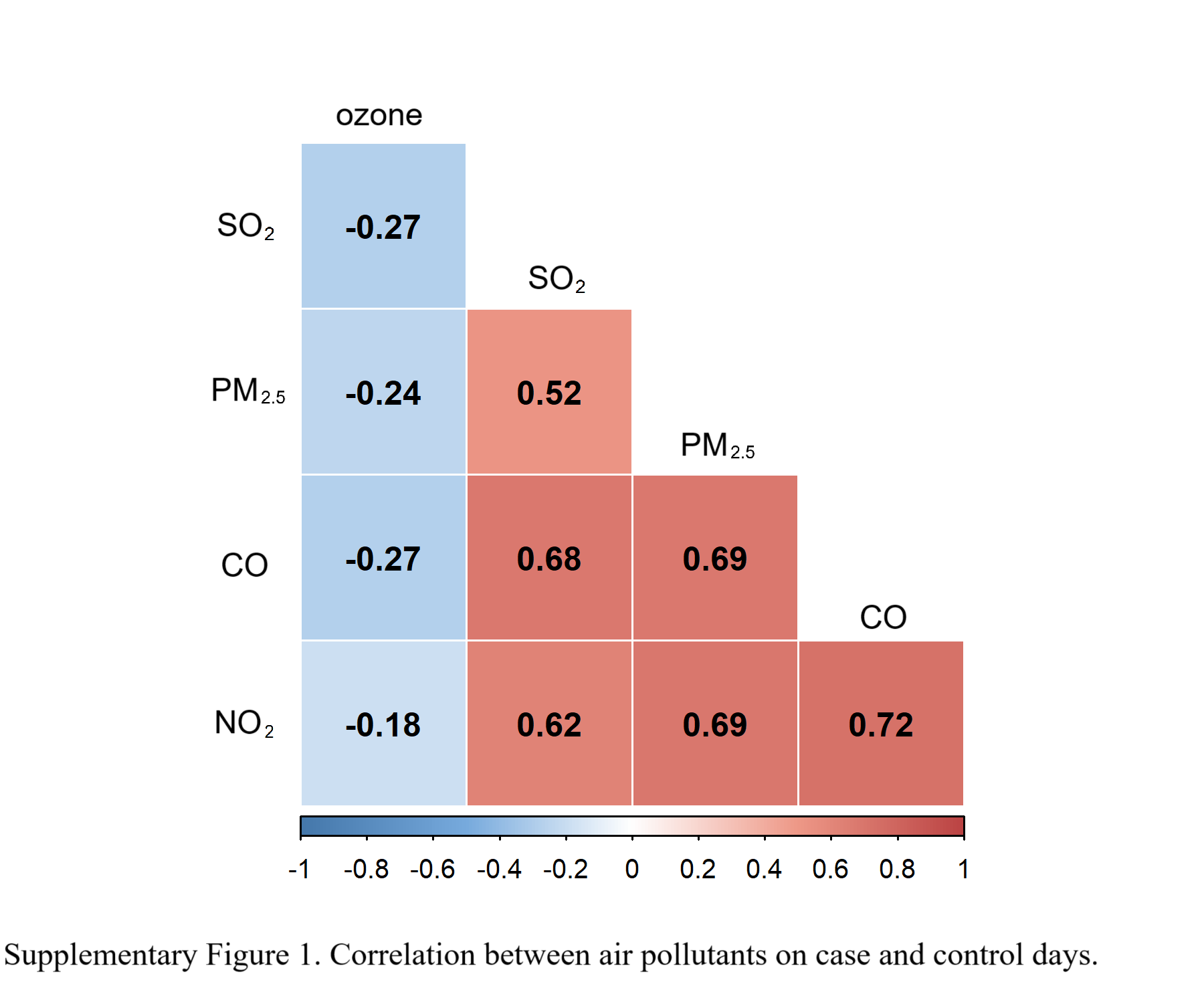

Supplement: Supplementary file 2 — Additional file 2: Supplementary Figure 1. Correlation between air pollutants on case and control days. [file ehpm-30-038-s002.png]

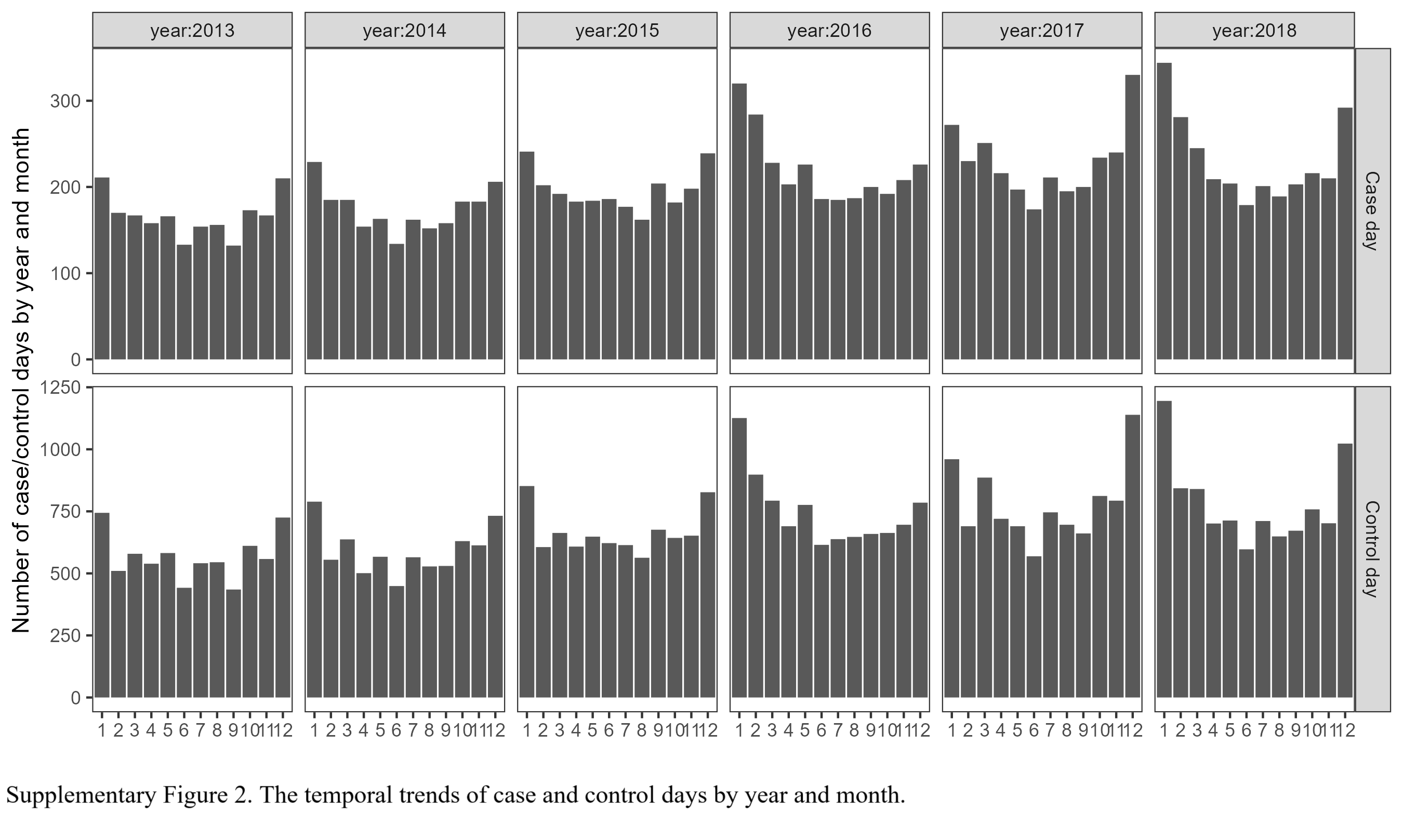

Supplement: Supplementary file 3 — Additional file 3: Supplementary Figure 2. The temporal trends of case and control days by year and month. [file ehpm-30-038-s003.png]

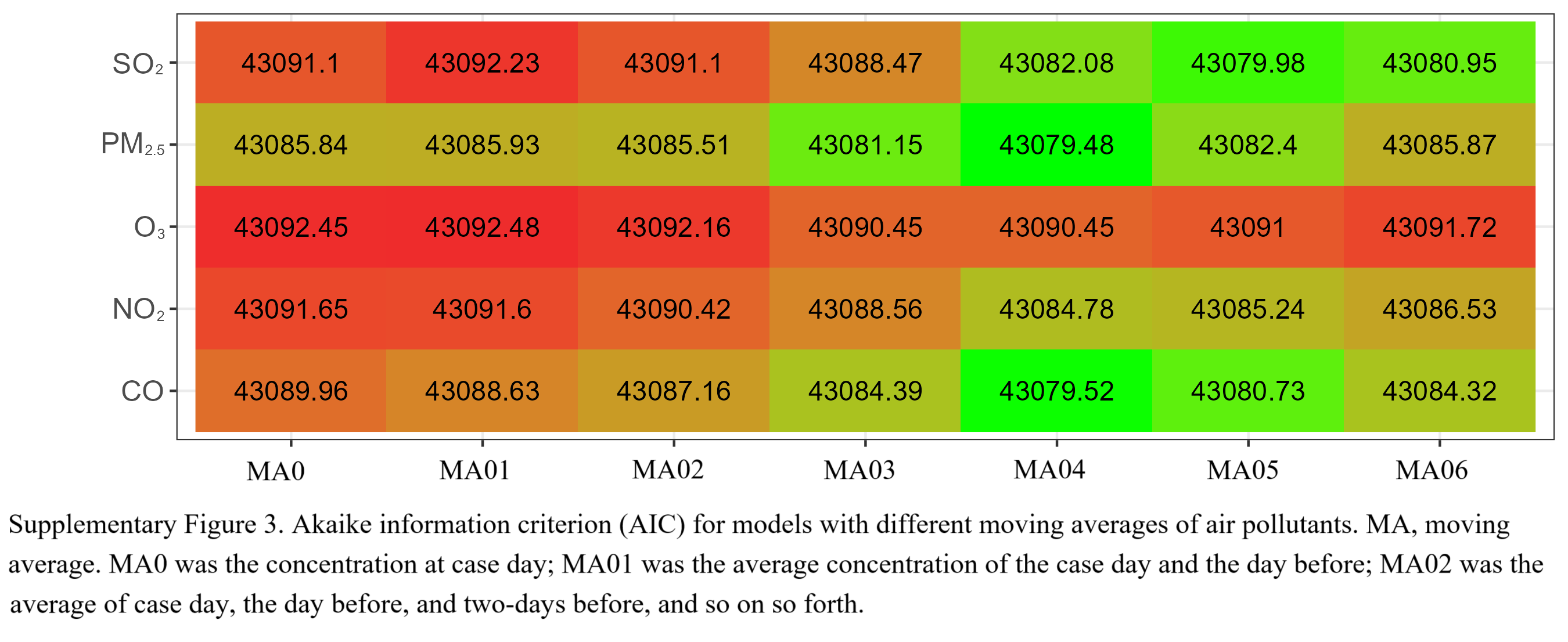

Supplement: Supplementary file 4 — Additional file 4: Supplementary Figure 3. Akaike information criterion (AIC) for models with different moving averages of air pollutants. [file ehpm-30-038-s004.png]

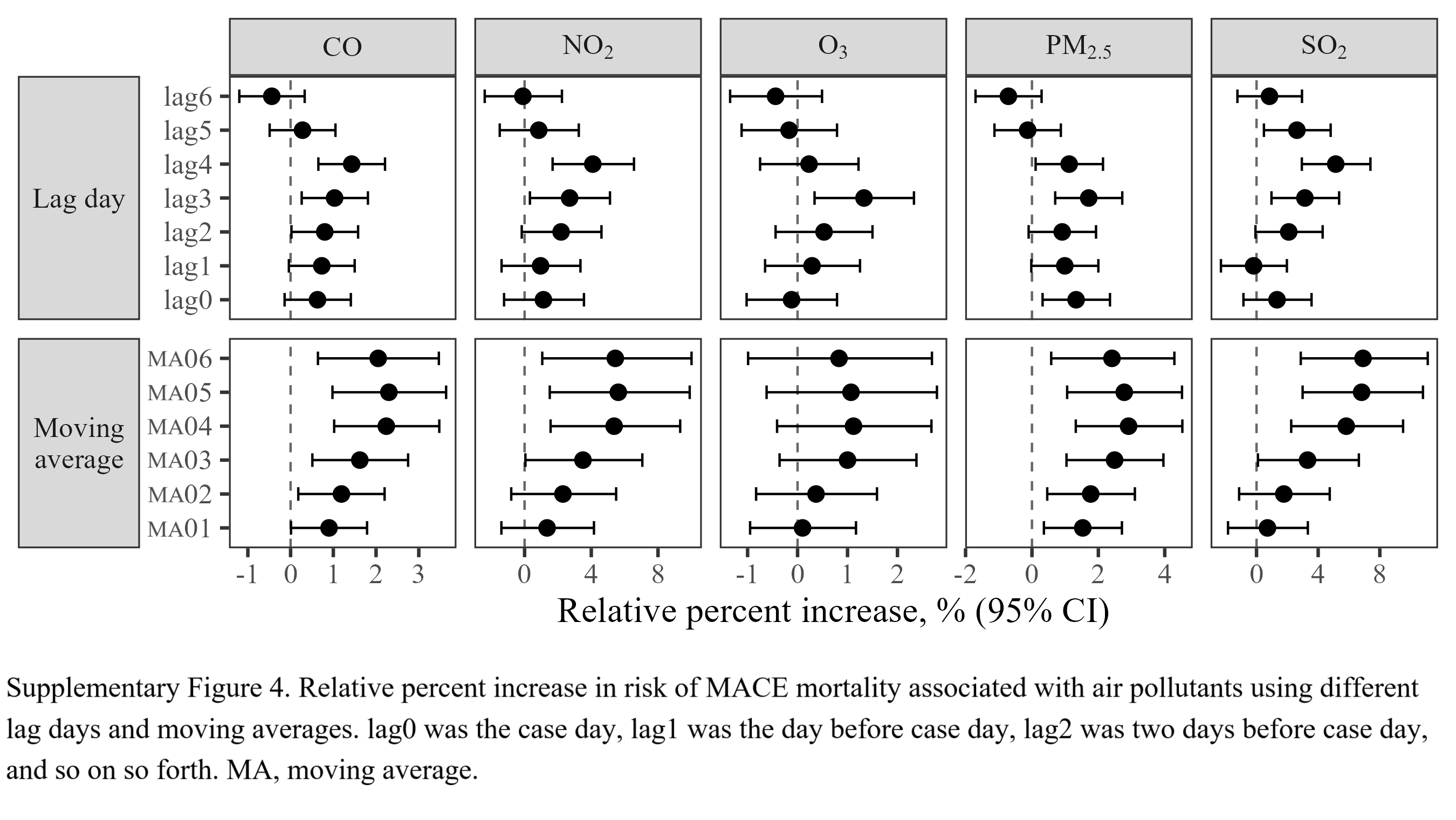

Supplement: Supplementary file 5 — Additional file 5: Supplementary Figure 4. Relative percent increase in risk of MACE mortality associated with air pollutants using different lag days and moving averages. [file ehpm-30-038-s005.png]
