# Supplementary material for: Association of short-term air pollution with risk of major adverse cardiovascular event mortality and modification effects of lifestyle in Chinese adults
Source: Environ Health Prev Med. 2025 May 13;30:38. doi: 10.1265/ehpm.24-00340 (PMC12086099; doi:10.1265/ehpm.24-00340)
Supplement: Supplementary file 6 — Additional file 6: Supplementary Table 1. Subgroup analyses of association between short-term exposure to air pollution and MACE mortality. Supplementary Table 2. Subgroup analyses of association stratified by socio-demographic factors. [file ehpm-30-038-s006.docx]

Supplementary Table 1. Subgroup analyses of association between short-term exposure to air pollution and MACE mortality. BMI:body mass index, overweight was defined as BMI>24; physical active was defined as in the upper half of metabolic equivalent of task in population with same age and sex; balanced diet was defined as daily consumption of fresh vegetable and fruit and weekly consumption of meat, fish, or poultry. Pval: p value for strata comparison from two-sample z test.

|  | **CO** | **pval** | **NO_2_** | **pval** | **O3** | **pval** | **PM_2.5_** | **pval** | **SO_2_** | **pval** |
| --- | --- | --- | --- | --- | --- | --- | --- | --- | --- | --- |
| **BMI** |  | 0.690 |  | 0.287 |  | 0.938 |  | 0.970 |  | 0.570 |
| overweight | 1.99 (0.14-3.88) |  | 8.15 (2.04-14.62) |  | 1.04 (-1.05-3.18) |  | 2.92 (0.47-5.44) |  | 5.54 (-0.37-11.80) |  |
| non-overweight | 2.49 (0.87-4.14) |  | 3.80 (-1.05-8.88) |  | 0.93 (-0.86-2.76) |  | 2.99 (0.90-5.11) |  | 7.85 (2.85-13.09) |  |
| **Drinking** |  | 0.110 |  | **0.019** |  | 0.418 |  | 0.815 |  | **0.016** |
| ever drink | 3.25 (1.50-5.02) |  | 10.25 (4.55-16.27) |  | 0.43 (-1.51-2.40) |  | 3.13 (0.84-5.47) |  | 12.01 (6.21-18.14) |  |
| never | 1.24 (-0.47-2.98) |  | 0.91 (-4.14-6.23) |  | 1.57 (-0.35-3.52) |  | 2.74 (0.54-4.99) |  | 2.28 (-2.75-7.57) |  |
| **Smoking** |  | 0.643 |  | 0.504 |  | 0.852 |  | 0.623 |  | 0.908 |
| ever smoke | 2.54 (0.82-4.28) |  | 6.70 (1.33-12.37) |  | 1.13 (-0.81-3.11) |  | 3.32 (1.09-5.60) |  | 6.54 (1.15-12.22) |  |
| never | 1.95 (0.22-3.72) |  | 4.06 (-1.28-9.68) |  | 0.87 (-1.04-2.81) |  | 2.51 (0.26-4.82) |  | 7.00 (1.63-12.65) |  |
| **Physical activity** |  | 0.421 |  | 0.254 |  | 0.106 |  | **0.015** |  | 0.722 |
| lower 50% | 2.58 (1.03-4.16) |  | 7.16 (2.14-12.43) |  | 1.97 (0.21-3.75) |  | 4.43 (2.40-6.51) |  | 7.28 (2.23-12.58) |  |
| upper 50% | 1.54 (-0.43-3.55) |  | 2.60 (-3.16-8.70) |  | -0.35 (-2.51-1.86) |  | 0.34 (-2.20-2.93) |  | 5.85 (0.07-11.96) |  |
| **Diet** |  | **0.013** |  | **0.007** |  | 0.081 |  | 0.158 |  | 0.493 |
| unbalanced diet | 2.61 (1.32-3.91) |  | 6.46 (2.44-10.63) |  | 0.49 (-0.95-1.96) |  | 3.21 (1.53-4.92) |  | 7.09 (3.13-11.21) |  |
| balanced diet | -1.06 (-4.87-2.91) |  | -7.84 (-19.38-5.35) |  | 5.05 (0.81-9.47) |  | 0.68 (-4.16-5.77) |  | 2.55 (-11.91-19.38) |  |
| **Healthy lifestyle** |  | 0.103 |  | **0.002** |  | 0.893 |  | 0.146 |  | **0.023** |
| lower 50% | 2.95 (1.43-4.49) |  | 9.96 (5.00-15.16) |  | 0.92 (-0.79-2.65) |  | 3.83 (1.84-5.87) |  | 10.24 (5.26-15.46) |  |
| upper 50% | 0.88 (-1.17-2.98) |  | -2.32 (-8.13-3.86) |  | 1.21 (-1.05-3.52) |  | 1.34 (-1.27-4.02) |  | 1.17 (-4.70-7.40) |  |

Supplementary Table 2. Subgroup analyses of association stratified by socio-demographic factors. Warm season included May to October, cold season included November to April. Pval: p value for strata comparison from two-sample z test.

|  | **CO** | **pval** | **NO_2_** | **pval** | **O3** | **pval** | **PM_2.5_** | **pval** | **SO_2_** | **pval** |
| --- | --- | --- | --- | --- | --- | --- | --- | --- | --- | --- |
| **Age** |  | 0.613 |  | 0.340 |  | 0.719 |  | 0.248 |  | 0.155 |
| <65yr | 1.51 (-1.57-4.68) |  | 0.91 (-8.52-11.30) |  | 0.42 (-3.10-4.07) |  | 0.75 (-3.18-4.84) |  | 0.89 (-7.64-10.21) |  |
| ≥65yr | 2.39 (1.06-3.73) |  | 6.14 (1.99-10.45) |  | 1.13 (-0.34-2.63) |  | 3.32 (1.59-5.09) |  | 8.09 (3.84-12.52) |  |
| **Education level** |  | 0.702 |  | 0.480 |  | 0.509 |  | 0.727 |  | 0.527 |
| ≤primary school | 2.38 (0.98-3.80) |  | 4.54 (0.29-8.97) |  | 1.24 (-0.38-2.89) |  | 3.07 (1.21-4.97) |  | 6.23 (1.98-10.65) |  |
| ≥middle school | 1.81 (-0.70-4.39) |  | 8.08 (-0.38-17.27) |  | 0.22 (-2.31-2.81) |  | 2.43 (-0.63-5.58) |  | 9.47 (0.77-18.92) |  |
| **Gender** |  | 0.555 |  | 0.545 |  | 0.774 |  | 0.535 |  | 0.698 |
| female | 1.84 (0.04-3.67) |  | 4.10 (-1.42-9.93) |  | 0.80 (-1.22-2.85) |  | 3.47 (1.10-5.90) |  | 6.01 (0.52-11.81) |  |
| male | 2.58 (0.93-4.27) |  | 6.50 (1.30-11.97) |  | 1.20 (-0.64-3.08) |  | 2.45 (0.32-4.63) |  | 7.56 (2.29-13.11) |  |
| **Marital status** |  | 0.774 |  | 0.595 |  | 0.996 |  | 0.617 |  | 0.072 |
| married | 2.15 (0.77-3.55) |  | 4.81 (0.51-9.28) |  | 1.00 (-0.54-2.56) |  | 2.69 (0.89-4.53) |  | 4.78 (0.49-9.24) |  |
| other | 2.58 (-0.01-5.24) |  | 7.37 (-0.69-16.09) |  | 1.00 (-1.92-4.01) |  | 3.68 (0.33-7.14) |  | 13.62 (5.34-22.56) |  |
| **Region** |  | 0.992 |  | 0.288 |  | **0.049** |  | 0.791 |  | **0.032** |
| rural | 2.43 (0.96-3.93) |  | 4.47 (0.09-9.05) |  | -0.33 (-2.10-1.47) |  | 3.10 (1.11-5.13) |  | 4.46 (0.31-8.77) |  |
| urban | 2.45 (0.22-4.72) |  | 9.55 (1.62-18.10) |  | 2.50 (0.35-4.70) |  | 3.56 (0.86-6.34) |  | 16.88 (6.83-27.87) |  |
| **Season** |  | 0.234 |  | **0.017** |  | 0.290 |  | 0.443 |  | 0.475 |
| cold | 2.89 (1.47-4.33) |  | 8.34 (3.78-13.09) |  | 0.11 (-2.05-2.32) |  | 3.73 (1.84-5.66) |  | 6.95 (2.60-11.48) |  |
| warm | 0.99 (-1.75-3.81) |  | -2.96 (-10.66-5.41) |  | 1.66 (-0.18-3.52) |  | 2.25 (-0.97-5.57) |  | 3.20 (-5.66-12.89) |  |
